# Effects of Nano-Composite Adsorbents on the Growth Performance, Serum Biochemistry, and Organ Weights of Broilers Fed with Aflatoxin-Contaminated Feed

**DOI:** 10.3390/toxins10090345

**Published:** 2018-08-27

**Authors:** Mookiah Saminathan, Jinap Selamat, Atena Abbasi Pirouz, Norhani Abdullah, Idrus Zulkifli

**Affiliations:** 1 Institute of Tropical Agriculture and Food Security, Universiti Putra Malaysia, Serdang 43400, Malaysia; saminathanupm@gmail.com (M.S.); norhani.biotech@gmail.com (N.A.); zulidrus@upm.edu.my (I.Z.); 2 Faculty of Food Science and Technology, Universiti Putra Malaysia, Serdang 43400, Malaysia; atena_pirouz@yahoo.com; 3 Department of Animal Science, Faculty of Agriculture, Universiti Putra Malaysia, Serdang 43400, Malaysia

**Keywords:** aflatoxicosis, broiler performance, blood profiles, organ weights, nano-composite adsorbent

## Abstract

The exploration of feed mycotoxin adsorbents to mitigate the adverse effects of mycotoxins on animals has received increasing attention over the last decade. The present study was conducted to assess the efficacy of nano-composite magnetic graphene oxide with chitosan (MGO-CTS) adsorbents against feed contaminated with ~20 ng/g (ppb) aflatoxin (AF). A total of three hundred 1-day-old chicks were randomly distributed into six dietary treatment groups, as follows: basal diet (broilers fed a diet with neither AF nor MGO-CTS added, T1), basal diet + 0.25% MGO-CTS (T2), basal diet + 0.50% MGO-CTS (T3), AF diet + 0.25% MGO-CTS (T4), AF diet + 0.50% MGO-CTS (T5), and AF diet (T6). The two inclusion levels (0.25 and 0.50%) of MGO-CTS significantly (*p* < 0.05) improved the growth performances and feed conversion ratios of the AF-treated chicks at 1–35 days of age, and the impact was more pronounced for 0.5% MGO-CTS. The AF intake markedly increased the relative weights of the liver and kidney, resulting in significant alterations in the serum biochemical parameters, such as albumins, alkaline phosphatase, and SGPT/alanine (ALT), at 35 days of age. However, the chickens fed 0.5% MGO-CTS with AF diets had apparent recovery or restoration of AF-induced organ lesions and aberrant serum profiles. A significant (*p* < 0.05) reduction in the total AFs was observed in the gastrointestinal tracts of the chickens fed 0.25% or 0.50% adsorbent in combination with AF feed (T4 and T5), with decreases of 28.9% and 53.5%, respectively, compared with that in the chickens fed an AF-contaminated diet (T6). The results of the study indicated that a higher concentration of MGO-CTS (0.50%) was effective in improving the overall performance of broiler chickens by preventing the adverse effects associated with aflatoxicosis.

## 1. Introduction

Mycotoxins are toxic low-molecular weight secondary active biological metabolites produced by filamentous fungi species that readily colonize crops. Aflatoxins (AFs) are highly toxic carcinogenic mycotoxins produced mainly by *Aspergillus flavus* and *A. parasiticus* [1]. The occurrence of AFs in animal feeds has received great concern because of the adverse effects on human and animal health via the contamination of animal feedstuffs and foods, as well as causing economic losses in livestock industries. The occurrence of AFs in animal feeds may reflect the climatic conditions on fodder crop, which can be retained in the relevant processed products (food/feed) and storage conditions of feedstuffs. Most of the feed for poultry supports *Aspergillus* spp. growth and AF formation [2]. AF B_1_, B_2_, G_1_, G_2_, and M_1_ (hepatic hydroxylated metabolite of AF B_1_) are the most common forms, but AFB_1_ is considered the most toxic [3].

The postulated adverse effects associated with AFs in animal feed on broiler chickens include a reduction in the growth performance and poor feed conversion [2], changes in the relative organ weights and immune-suppression [4], increased mortality [5], causing liver and kidney damage [6], and enhanced susceptibility to infectious diseases [7]. The palm kernel cake (PKC) has been incorporated as a cost-effective feed ingredient for poultry feed. PKC is abundantly available in palm oil-producing countries. The AF contamination of PKC in poultry feed due to inappropriate handling during the PKC production and storage could increase health risks and decrease the production of poultry [8]. The European Commission (EC) has prescribed the maximum contamination level for AFB_1_ to be 0.02 mg/kg in the feedstuffs of poultry to protect these animals from health risks and to prevent mycotoxin transmission into meat and meat products [9]. Furthermore, animal trials examining the efficiency of potential AFs binders for use in feed have been conducted at AFs levels not exceeding maximum EC contamination regulation, in order to minimize the detrimental effects of these contaminants on host biological systems [2,6,10].

Numerous strategies, such as thermal inactivation, microbial degradation, physical separation, irradiation, and treatment with a variety of chemicals have been proposed to detoxify the mycotoxin-contaminated feedstuffs. Among the most frequently used chemical adsorbents to bind, absorb, or degrade toxins so as to alleviate the toxin effect from animal feedstuffs are activated charcoal, aluminosilicates (zeolites, hydrated sodium calcium aluminosilicate, and clays), and montmorillonites treated with organic cations, sodium bentonite, and chitosan polymers [6]. The efficacy of adsorbents has been examined in vivo to determine their ability to bind toxins and to prevent health risks in broiler chickens, by observing their growth performance, hematological and serum biochemical parameters, and liver morphology [6,11,12]. However, most studies focused on AFs, particularly aflatoxin B_1_ (AFB_1_), as it is highly toxigenic compared with other mycotoxins.

The magnetic graphene oxide (MGO) is a new class of nanostructured multifunctional nanocomposite materials. It is synthesized from iron oxide nanostructures and graphene oxide (GO). Unlike a GO compound, MGO nanoparticles have been widely used in environmental remediation because of their ease of separation from aqueous mixtures [13]. Hence, MGO-based nano-composites have been used as adsorbents in wastewater treatment to remove heavy metals and radionuclides from contaminated solutions [14]. MGO belongs to a class of electron-rich carbon adsorbents with a large delocalized π-electron system, which may show promising applications in the removal of mycotoxins [15]. Recently, we developed an adsorbent MGO with chitosan (MGO-CTS), which shows a marked increase in the absorption of AFs, ochratoxin A, and zearalenone from PKC [15]. This novel adsorbent involves the transformation of surface properties by exchanging structural charge-balance cations with high-molecular weight quaternary amines and increasing the amount of carbon, resulting in an increased adsorption capacity for removing multi–mycotoxins. MGO-CTS can primarily act as an adsorbent to bind AFs and to reduce mycotoxin effects on broilers. Broiler chickens fed diets naturally contaminated with AFs could have reduced weight gain, altered immune systems, and increased organ damage, whereas the use of MGO-CTS might mitigate these adverse effects. The present study was conducted to investigate the AF-binding ability and the protective efficacy of MGO-CTS at two different levels on the growth performance, serum biochemistry profiles, relative organ weights, liver histopathologies, and AF concentrations in the duodenal content of broilers fed diets contaminated with AFs at levels not exceeding the EU recommendations.

## 2. Results

### 2.1. Growth Performance

The effects of dietary treatments on the body weights of the broilers are shown in Table 1. The broiler body weight did not differ between the experimental treatments during week 1, 2, and 3. Broilers receiving diets with a mixture of AF-contaminated feed and 0.25% or 0.50% MGO-CT adsorbents (treatments T4 and T5, respectively) showed lower body weights at the end of the fourth week compared with those fed the basal diet (T1) or uncontaminated diets with adsorbents (T2 and T3). However, in the fifth week (end of the experimental period), no significant (*p* > 0.05) differences in body weight were observed between the diets with a mixture of AF-contaminated, and 0.25% or 0.50% MGO-CT adsorbents (T4 and T5), and basal diet (T1), but these values were significantly (*p* < 0.05) higher than those for the contaminated diet (T6). There was no difference in the levels of the adsorbents added, with respect to their effects on weight, regardless of whether the chickens were administered feed without (T2 and T3) or with (T4 and T5) AFs. 

The feed intake was not significantly different among all of the dietary treatment groups from 1 to 21 days of age (Table 2). However, the feed intake in the chickens fed a diet with 0.25% adsorbent (T4) was significantly (*p* < 0.05) higher than that in the broiler chickens fed contaminated diets (T6), but not significantly different (*p* > 0.05) from those of the other treatments, from 22 to 35 days of age and from 1 to 35 days of age. For the first three weeks, the body weight gain was significantly (*p* < 0.05) higher in the chickens that were fed the basal diet (T1) and the mixture of the contaminated diet with 0.5% of adsorbent (T5), but these values were not significantly different (*p* > 0.05) from those fed the uncontaminated diets with 0.25% and 0.5% binders (T2 and T3), and the contaminated diet with 0.25% adsorbent (T4), when compared to the effects of the AF-contaminated diet (Table 2). From 22 to 35 days of age and from 1 to 35 days of age (entire experimental period), significant (*p* < 0.05) improvements in body weight gains were observed in the broiler chickens that were fed the basal diet, uncontaminated, and contaminated diets, regardless of the level of binder (T2, T3, T4, and T5 respectively), when compared to broiler chickens fed a contaminated diet (T6).

The feed conversion ratio (FCR) value of the broiler chickens was significantly (*p* < 0.05) lower in the basal diet (T1) when compared to the broiler chickens fed a contaminated diet (T6) from 1 to 21 days of age, but was not significantly different from that of the treatments of T2, T3, T4, and T5 (Table 2). From 22 to 35 days of age, a significantly (*p* < 0.05) lower FCR was observed in the broiler chickens fed uncontaminated diets with a 0.25% adsorbent (T2) diet, when compared to that in the chickens fed contaminated diets with 0.25% adsorbent (T4) (Table 2). Overall (1 to 35 days of age), the broiler chickens receiving all of the treatment diets, except for the contaminated diet with 0.25% adsorbent (T4), showed significantly (*p* < 0.05) lower FCR values compared with that of the chickens receiving the contaminated diet (T6) treatment. Although mortality was numerically high in the contaminated diet (T6) treatment group (10%), the Pearson chi-square test showed no difference (*p* > 0.05) in the mortality rate between the dietary treatments.

### 2.2. Broiler Chicken Organ Weights

The relative organ weights were calculated in g/100 g of the body weight of chickens, and the results are shown in Table 3. The relative weights of the gizzard, liver, heart, pancreas, proventriculus, and bursa of Fabricius did not statistically differ (*p* > 0.05) between the experimental treatments. However, the kidney weight was significantly higher for all of the treatments, when compared with that of the basal diet (T1) treatment at 35 days of age (Table 3). At 35 days of age, the chickens supplemented with a contaminated diet (T6) had significantly (*p* < 0.05) higher spleen weights than the other treatment groups, except for those that were fed contaminated diets with a 0.25% binder (T4). 

### 2.3. Biochemical and Haematological Parameters

The effects of dietary treatments on the serum biochemical parameters of the broilers are shown in Table 4. Haematocrit did not significantly differ (*p* = 0.546) between the treatments and ranged between 26–32%. The concentrations of cholesterol, total proteins, globulins, urea nitrogen, γ-GT^3^, and the albumin/globulin ratio were also not significantly different (*p* > 0.05) among the treatments. Of the two transaminases examined, only alanine (ALT) differed between the treatments, which was significantly lower (*p* < 0.05) in the broiler chickens fed an uncontaminated diet with 0.5% adsorbent, a contaminated diet with 0.25% adsorbent (T4), and a contaminated diet (T6), compared with those fed a basal diet (T1). The serum albumin levels were significantly reduced (*p* < 0.05) in the broiler chickens fed contaminated diets with different levels of adsorbent added (T4 and T5) and a contaminated diet (T6), when compared to the broiler chickens fed a basal diet (T1). The addition of contaminated diet with 0.25% adsorbent (T4) and a contaminated diet (T6) significantly decreased (*p* < 0.05) the levels of alkaline phosphatase, compared with that in the chicks fed a basal diet alone (T1), whereas this value in the chicks that were fed a contaminated diet with 0.5% adsorbent (T5) was not significantly different (*p* > 0.05) from that in the chicks fed a basal diet.

### 2.4. Levels of Aflatoxins Recovered from Duodenal Contents

**Figure 1:**
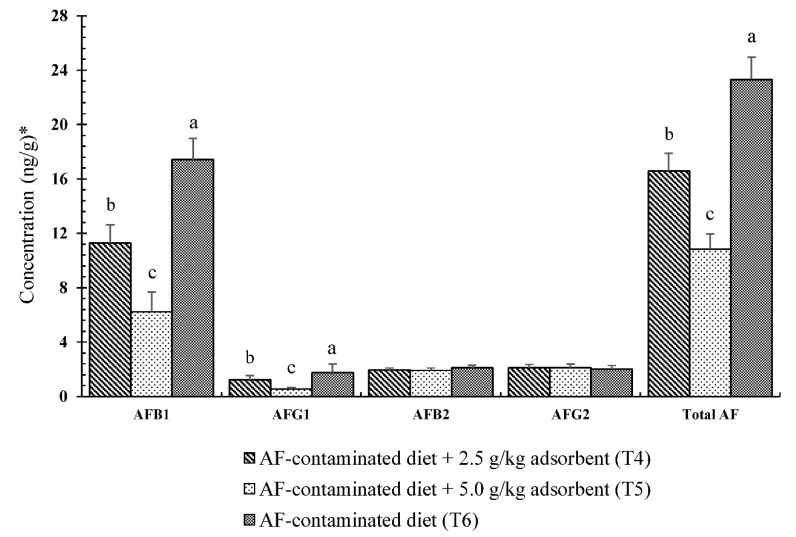
Effects of magnetic graphene oxide with chitosan (MGO-CTS) adsorbent on aflatoxin B_1_ (AFB_1_), aflatoxin B_2_ (AFB_2_), aflatoxin G_1_ (AFG_1_), aflatoxin G_2_ (AFG_2_), and the total AF concentrations recovered from the duodenal contents of the broilers fed palm kernel cake (PKC)-based feed naturally contaminated with AFs. Bars show the mean of six replicates (*n* = 6) and the error bars are the standard error of the mean. Within the same type of AF, bars with different letters (a, b, c) differ significantly (*p* < 0.05). * AFB_1_, AFB_2_, AFG_1_, and AFG_2_ were not detected in the duodenal contents of the broilers under the T1 (basal diet), T2 (basal diet + 2.5 g/kg adsorbent), and T3 (basal diet + 5.0 g/kg adsorbent) treatments. Total AF = AFB_1_ + AFG_1_ + AFB_2_ + AFG_2_.

The amounts of AFs recovered from the duodenal contents are shown in Figure 1. AFB_1_, AFB_2_, AFG_1_, and AFG_1_ were not detected in the duodenal contents of the broiler chickens that were fed diets of T1 (basal diet) and uncontaminated diets with 0.25% or 0.50% binders (treatments T2 and T3, respectively) (detection limits were 0.03 ng/g for B_1_ and G_1_, and 0.05 ng/g for B_2_ and G_2_). A significant (*p* < 0.05) reduction in the AFB_1_ concentration was observed in the treatments with contaminated diets supplemented with adsorbents (T4 and T5), with a decreases of 35.24% (6.14 ng/g) and 64.15% (11.18 ng/g), respectively, when compared with that of the contaminated diets (T6). The concentrations of AFG_1_ in all of the treatment groups showed similar trends to those of AFB_1_. Treatment T5, which had a high level of adsorbent (0.5%) in the AF-contaminated diet, induced the greatest decrease (69.25%) in AFG_1_, followed by the AF-contaminated diets with 0.25% adsorbent (T4), with a reduction of 31.31%, compared with that of the contaminated diet (T6). There were no significant differences (*p* > 0.05) in the AFB_2_ and AFG_2_ concentrations among all treatment groups. Chicks fed a contaminated diet with 0.5% adsorbent (T5) showed the highest decrease in total AF (53.5%), followed by AF-contaminated diets with 0.25% adsorbent (T4), with a reduction of 28.9%, compared with that of the contaminated diet (T6). 

### 2.5. Hepatic Histopathology

No apparent histological changes or alterations were observed in the liver sample tissues of the broiler chickens from the control and treatment groups, presumably because of the presence of AFs at low levels in the diet.

## 3. Discussion 

PKC, commonly used as an economic ingredient in feed for poultry, is highly susceptible to aflatoxicosis because of improper handling and storage. The concomitant use of AF-contaminated feedstuff could result in adverse consequences on the growth and metabolism of broiler chickens. To overcome the aflatoxicosis, MGO-CTS adsorbents comprising unique plate structures, large specific surface areas, excellent stability, and rich functionalities can be used as multi-mycotoxin binders to reduce the negative effects on poultry [15]. The major advantages of this adsorbent include its stability in gastrointestinal fluids, as it might not be absorbed via the gastrointestinal tract, and easy administration by inclusion into animal feed.

In the present study, two levels of MGO-CTS adsorbent (0.25 and 0.5%) treatments were applied to measure the growth performance of broiler chickens fed with or without AF-contaminated feed, where a known amount of mycotoxin was reacted with a known amount of adsorbent. The results indicated that the broiler chickens that were fed a basal (control) diet as well as AF-contaminated diets with the two levels of MGO-CTS adsorbent, performed equally well in live mass dynamics (ranged from 1.97 kg to 2.02 kg) compared to the chickens that were fed AF-contaminated diets (1.85 kg) at the end of the experimental period. This finding suggests that the two levels of MGO-CTS adsorbents that were examined may limit the adverse effects of the AFs present in feed at levels not exceeding the EU limits. Similar results of weight gain in the chickens that were fed either a basal diet or with non-contaminated feed with the inclusion of adsorbents, further confirm the safety of the reaction products and the safeguarding of the nutritional characteristics of the treated feeds.

Reduction in the growth performance of broiler chickens fed AF-contaminated diets may be attributed to the ability of the AFs to inhibit metabolism [16] and limit body protein synthesis by competing with phenylalanine for binding sites on the phenylalanine-transfer RNA synthetase [17]. Thus, it is not surprising that, in the present study, the weight gains and feed conversion rations (FCRs) of the broiler chickens were adversely affected by the AF diets. Similar effects have previously been reported [18] in chickens fed diets with 0.03 ppm (mg/kg) AFs. However, the presence of MGO-CTS in contaminated diets, indicating the efficacy and optimal amount of adsorbent in the combined feed for the detoxification of mycotoxins, resulted in an improved feed conversion of animal feed to meat. The feed intake was not significantly affected, as such, the AF contamination levels in the broilers that were presumed to have similar nutrition values as the basal (control) diet.

Aflatoxicosis in broilers can cause an abnormality of specified bodily organs, such as the liver and kidneys, by increasing the relative weights of these organs [19]. The liver is considered the primary target organ for AFs because AFs mainly accumulate in the liver after absorption for detoxification [20]. An increase in the liver weights of the broilers may be due to the accumulation of lipids and the inhibition of lipid transport in the liver, influenced by the presence of mycotoxins. However, the changes in the liver weight appear to be associated with the AF level in the diet. In the present study, no significant difference in the liver weight was found in the broilers fed AFs at levels higher than 20 μg/kg. This finding is similar to that of Ma, et al. [21], who reported that the relative liver weights of the chickens were not altered in the broilers consuming diets containing AF levels of 50–100 μg/kg. Therefore, we infer that perhaps this variable, the weight of the liver, is not sensitive to low-dose AFs. In the present study, the relative weights of the liver, heart, gizzard, and bursa of Fabricius of the broilers fed the non-contaminated and contaminated diets with or without adsorbents were similar to those found by Pasha et al. [2] in the chickens receiving AF diets with adsorbents. The similar results were observed for the weight of the pancreas [10]. However, the kidney weight was increased by the AF-contaminated diets (*p* < 0.05), and the inclusion of the two levels of adsorbent in both the uncontaminated and contaminated diets failed to restore the normal proportion of the kidney compared to that of the chickens that were fed the control diet. An increase in the relative weight of a major excretory organ in a chicken fed an uncontaminated diet may be related to the absorbent volume in the diet or the rheological properties of the adsorbent. Similarly, Aravind et al. [22] reported that the addition of lower concentrations of esterified glucomannan (E-GM) toxin binder (0.05% of diet) to an uncontaminated broiler diet increased the kidney weight. The spleen weight was considered a sensitive indicator of immunotoxicity (immune stimulation or depletion), stress, and physiologic perturbations. In the present study, the relative weight of the spleen was increased by the AF-contaminated diet (*p* < 0.05) and could be restored to normal proportions, only by the addition of 0.5% MGO-CT adsorbent. The normal relative weight of the spleen was observed by the inclusion of a higher level of adsorbent (0.5%) in the AF diet, indicating that the adsorbent had protective effects against the lesions induced by AFs.

Previous studies have confirmed that chickens suffering from experimental aflatoxicosis are hypoproteinaemic, a condition in which there is a deficiency in the total protein concentration in the plasma or serum [23]. The serum ALT and aspartate (AST) activities [24], as well as the total protein, albumins, globulin, and urea concentrations [25] have been described as valuable parameters of hepatic injury and function. In the present study, the haematocrit, the ratio of the volume of red blood cells to the total volume of blood, ranged between 26 and 32% within the normal range (22–35%). These results confirmed that the total protein, cholesterol level, globulin content, urea concentration, and γ-glutamyltransferase levels are mildly affected in the AF-fed chickens. These non-significant effects are most likely due to their low concentration AFs (20 ppb, EU maximum level of AFs in animal feed) and potentially due to the action of the adsorbent.

Nevertheless, the ALT concentration was lower in the presence of AFs with or without adsorbents; however, the levels were generally below those indicating hepatotoxicity [26]. Similar results were observed by Che et al. [12] in broilers fed diets with mould-contaminated feed, but without adsorbent, and a decrease in the ALT was observed. In the present study, the AFs in the broiler feed with or without adsorbent led to a marked decrease in the chicken serum albumin levels. Corresponding results were reported in the broiler chickens fed 0.11 mg/AFs kg feed [10]. This result suggested that the serum albumin might be a sensitive index for mycotoxin effect investigations. Generally, AF adducts alter protein synthesis and bind cellular macromolecules of the liver, reducing the albumin levels in the serum [27]. In the present study, the AF residues in the feed escaping from the MGO-CTS adsorption could enter the blood circulatory system and adversely affect the albumin levels. 

In a previous study, the pseudo-first and pseudo-second order kinetics models most effectively explained the MGO-CTS adsorption capacity of multi-mycotoxins (AFB_1_, OTA, and ZEA, unpublished data). Hence, the simultaneous binding affinity of multi-mycotoxins to MGO-CTS may be related to the strength and number of bonds formed between the adsorbent and mycotoxin molecules. The present study revealed that both levels of MGO-CTS adsorbents examined exhibited a binding capacity for AFB_1_ and AFG_1_ in gastrointestinal tract contents, and the impact was more pronounced for the higher binder volume (0.5%). Nevertheless, high amounts of adsorbent may have high surface areas for increased mycotoxin binding. Generally, AFs are chemically hydrophilic aromatic molecules with a high affinity for binding planar surfaces [28]. The possible mechanism of action of MGO-CTS was by binding the AF in the gut to form stable MGO-aflatoxin complexes, which were excreted from the body through the cloaca, reducing its absorption in the gastrointestinal tract of the chickens. Pirouz et al. [15] suggested that the reduction of mycotoxins by MGO-CTS involves two adsorption mechanisms. Particularly, the π–π stacking noncovalent interaction between the aromatic ring of mycotoxins and the GO basal planes has been considered a fundamental factor in binding. Moreover, the electrostatic attraction between the negatively charged analytes (COO−) and the positively charged iron ions (Fe^+2^ and Fe^+3^) of MGO accelerated the electron transfer between the materials. Ferric and ferrous cations present in the MGO may form coordinated and electrostatic bonds with the β-dicarbonyl system of the AFs, explaining the high absorbance capacity of the MGO-CT nano-composite. 

## 4. Conclusions

In conclusion, this study showed that the application of the two levels of MGO-CTS adsorbents could limit the adverse effects of the AFs present in the feed (at levels not exceeding the EU limits), thus maintaining the optimal broiler performance, relative organ weights, and serum profiles of the broiler chickens, compared with those of the chickens fed control diets, and the impact was more pronounced for the high level of MGO-CTS (0.5%). The inclusion of a higher volume of MGO-CTS in the feed also revealed that more than half of the AFs were absorbed from the contaminated feed in the gastrointestinal tracts. The alleviation of mycotoxins in the broilers’ feed containing MGO-CTS could mitigate the negative effects of the mycotoxin contaminated feed, which could subsequently increase the safety of poultry products. 

## 5. Materials and Methods

### 5.1. Aflatoxin Production in PKC

Aflatoxins (AFB_1_, AFB_2_, AFG_1_, and AFG_2_) were produced by the fermentation of PKC, using the *Aspergillus parasiticus* FRR 2999 strain, as described by Pappas et al. [6], with minor modifications. PKC was inoculated with *Aspergillus* conidia (approximately 10^9^ conidia mL^−1^) via dispersion and placed in Erlenmeyer flasks. After culturing for 14 days at room temperature in the dark, the flasks were autoclaved, and the material was dried at 30 °C for two days. The concentration of AFs in the PKC was determined by liquid chromatography-tandem mass spectrometry (LC-MS/MS), as described in the following section.

### 5.2. Sample Preparation and Determination of Aflatoxin in the Feed 

The extraction of AFs from the feed samples of each treatment were performed according to the method of the Association of Official Analytical Chemists [29], with modification by Afsah-Hejri et al. [30]. Specifically, 25 g of the feed samples were blended with 5 g of NaCl in 125 mL of methanol/water (70:30, *v*/*v*) for 2 min, using a Waring blender (Vicam, Milford, MA, USA). The homogenate was diluted with 30 mL of dH_2_O and filtered using a 24 cm Ø fluted filter paper (Vicam, Milford, MA, USA). The supernatant was filtered again through an 11 cm Ø glass microfiber filter (Vicam, Milford, MA, USA). Then, 15 mL of the filtrate was passed through the immunoaffinity column (IAC) (Aflatest; Vicam, Milford, MA, USA) at a flow rate of 1 mL/min, to bind and purify the AFs [31]. The IAC was then washed twice with 10 mL of dH_2_O, followed by the elution of AFs with 1 mL of absolute methanol. The eluent was diluted with 1 mL of dH_2_O and stored in high-performance liquid chromatography (HPLC) vials until further analysis.

The purified AFs were quantified using a reverse-phase high-performance liquid chromatography (RP-HPLC) system (Waters 600, New York, NY, USA) consisting of a fluorescence detector (Waters 2475, New York, NY, USA) and a post-column photochemical reactor enhancement detection system (PHRED) (Aura Industries Inc., New York, NY, USA) equipped with a 250 mm × 4.6 mm i.d., 5 µ, C_18_ analytical column (Waters, New York, NY, USA). The wavelengths of the fluorescence detection were 360 nm for excitation and 440 nm for emission. The sample injection volume was 20 µL, with an isocratic mode mobile phase solvent composition of H_2_O:MeOH:CH_3_CN (55:35:10 *v*/*v*/*v*), at a flow rate of 0.6 mL/min.

The standard curves for AFB_1_, AFB_2_, AFG_1_, and AFG_2_ were constructed with seven concentrations of 2, 4, 6, 10, 25, 50, and 100 ng/g. The R^2^ values obtained from all of the four standard curves were >0.995. The aflatoxin retention times were 17.8 min for AFG_2_, 20.7 min for AFG_1_, 23.4 min for AFB_2_, and 27.6 min for AFB_1_. The aflatoxin peaks were recorded and integrated using Empower 2 Chromatography Data software (Waters, New York, NY, USA). The detection limits were 0.03 ng/g for B_1_ and G_1_ and 0.05 ng/g for B_2_ and G_2_.

### 5.3. Synthesis of Nano-Composite Modified Adsorbents 

The modified adsorbents were synthesized according to Pirouz, et al. [15]. First, graphene oxide (GO) was synthesized using Hummer’s method [32], with some modifications according to Marcano, et al. [33]. Subsequently, magnetic GO (MGO) was prepared by the co–precipitation of ferrous (Fe^2+^) and ferric (Fe^3+^) ions on GO sheets, according to the method of Prakash, et al. [34]. Finally, the novel MGO-CTS nano-composite was prepared by dissolving 0.4 g of powdered chitosan in 20 mL of acetic acid solution (2% *v*/*v*), under ultrasonic stirring for 2 h at room temperature. Then, 0.3 g of MGO was added to the prepared solution and stirred for 90 min in a water bath at 50 °C. The pH of the mixture was then adjusted to 9–10 with NaOH (0.1 mol/L), and was incubated in a water bath for another 60 min at 80 °C, followed by drying in a vacuum oven at 50 °C. The MGO-CTS composites were ground into fine powder at 75–125 mm, after sieving and stored in airtight containers until further use. 

### 5.4. Broiler Chickens and Diets

Three hundred 1-day-old male broiler chicks (Cobb 500) obtained from a local commercial hatchery were used in the present study. The broiler chicks were randomly assigned to six dietary treatments with 50 broiler chicks in each group. The basal diet (antibiotic-free) and treatment diets were formulated to meet the nutrient requirements recommended by the National Research Council (NRC) [35] for starter (1–21 days; 20.8% CP, crude protein; 2.99 Mcal/kg) and grower (22–35 days; 18.4% CP, 2.94 Mcal/kg) periods. The compositions of the diets are shown in Table 5. The following dietary treatments were used: (T1) basal diet (broilers were fed a diet with neither mycotoxins nor adsorbent added), (T2) basal diet + 2.5 g/kg adsorbent, (T3) basal diet + 5.0 g/kg adsorbent, (T4) basal diet + AF-contaminated diet + 2.5 g/kg adsorbent, (T5) AF-contaminated diet + 5.0 g/kg adsorbent, and (T6) AF- contaminated diet (Table 6). The necessary quantities from each of the three parts of the AF-contaminated PKC were incorporated into a basal corn–soybean meal broiler chicken diet (i.e., T4, T5, and T6) to provide the desired level of AFs, which did not exceed the EU maximum. The broiler chicks were reared in three-tiered battery cages (0.9 × 1.2 × 1.75 m, length × width × height), with a raised wire-netted floor in an open house under natural tropical conditions (temperatures of 24–33 °C, relative humidity of 80–100%), with 12 h of natural daylight and artificial lighting during 12 h of darkness. Five cages containing 10 broiler chicks each were used as replicates for each treatment.

The broiler chicks were brooded with a 100 W bulb for 14 days. Water and feed in mash form were provided ad libitum. At weekly intervals, the body weights of the broilers were weighed and recorded, and the body weight gain was calculated at 1, 21, and 35 days of age. The feed intake was measured weekly, and the feed consumption and the feed to gain ratio (FCR) were calculated for 1–21, 22–35, and 1–35 days of age. The broilers were inspected daily, and mortality was recorded. The total mortality was calculated as the number of broiler chickens that died throughout the study compared to the initial number of broiler chickens placed. The temperature and relative humidity of the surrounding environment were recorded daily. All of the animal management and sampling procedures were approved by the Universiti Putra Malaysia’s Animal Care and Use Committee (UPM/IACUC/AUP-R048/2018, 14 August 2018).

### 5.5. Sampling Procedures

At 35 days of age, 10 broiler chickens (two broiler chicken from each replicate cage) from each treatment were weighed and sacrificed, and the blood was collected in non-heparinized blood collection tubes in order to obtain the serum for the determination of aspartate aminotransferase (AST, IU/L), alanine aminotransferase (ALT, IU/L), urea (mmol/L), γ-glutamyltransferase (γ-GT, IU/L), alkaline phosphatase (IU/L), cholesterol (mmol/L), total proteins (g/L), albumins (g/L), globulins (g/L), albumins/globulins, and haematocrit (%). The samples were analysed using an automated ABX Pentra 400 Bench Top Analyser (Horiba-ABX, Montpellier, France). In addition, the carcasses of the sacrificed chickens were immediately opened, and the gizzard, liver, heart, pancreas, spleen, kidney, bursa of Fabricius, and proventriculus were removed; their weights were expressed in g/100 g of body weight.

### 5.6. Hepatic Morphology

At 35 days of age, the livers of the broiler chickens (which were excised to measure the relative organ weight) were used for hepatic morphology analysis. Part of the liver sample (the tip of the right lobe) was fixed in 10% neutral-buffered formalin solution. The fixed tissue was dehydrated in graded alcohol, cleared in xylene, and embedded in paraffin. Thin sections (3–5 μm) were obtained and subsequently stained with haematoxylin and eosin (H&E) for histopathological examination using optical microscopy (Olympus Optical Company, Tokyo, Japan), according to Bancroft and Gamble [36]. 

### 5.7. Aflatoxins Recovered from Duodenal Content 

The duodenal contents (from the ventriculus to pancreo-biliary ducts) of two broiler chickens from each treatment replicate cage were collected, pooled, and homogenized in the same tube. The samples were immediately stored at −80 °C prior to freeze-drying. Approximately 25 g of freeze-dried duodenal contents were used for AF extraction. The sample preparation and RP-HPLC analysis method for the determination of the AF levels in the duodenal contents was the same as that described above for the determination of AF in the feed samples.

### 5.8. Statistical Analysis

The statistical analysis of the data was performed as a completely randomised design using the PROC GLM general factorial ANOVA procedure, using SAS version 9.2 (SAS Institute Inc., Cary, NC, USA). All of the performance variables (body weight, feed intake, body weight gain, and FCR) for each growth interval, and the parameters, determined once, such as the relative organ weight, biochemical and hematological parameters, and duodenal AF content, were analysed by ANOVA using treatment as the main factor. The GLM model that was used was Y*ijk* = µ + T*i* + ε*ij* + δ*ijk*, where Y*ijk* is the dependent observation; µ is the mean effect; T*i* is the *i*th treatment effect; ε*ij* is the random error; and δ*ijk* is the sampling error. Statistically significant effects were further analysed and means were compared using Duncan’s new multiple range test. The percentage data of mortality was analysed using Pearson chi-square test to identify the significant differences between the treatments. Statistical significance was determined at *p* ≤ 0.05.

## Figures and Tables

**Table 1:** The effect of two level of magnetic graphene oxide with chitosan (MGO-CTS) and aflatoxin (AF)-contaminated diets on broiler body weight (g).

Age (d)	Broiler Weight (g)							
	T1	T2	T3	T4	T5	T6	SEM	*p*-Value
0	45	45	47	47	45	44	0.27	0.132
7	188	185	182	185	189	180	1.59	0.898
14	487	486	487	487	487	472	2.96	0.091
21	886 ^a^	849 ^b,c^	870 ^a,b^	878 ^a^	872 ^a,b^	836 ^c^	5.81	<0.05
28	1463 ^a^	1457 ^a^	1432 ^a,b^	1390 ^c^	1403 ^b,c^	1337 ^d^	9.54	<0.05
35	2065 ^a^	2033 ^a^	2067 ^a^	2014 ^a^	2047 ^a^	1895 ^b^	12.50	<0.05

Data represent mean values of five replicate cages, each with 10 chickens. ^a–d^ Means within a row with no common superscript differ significantly (*p* < 0.05). SEM—standard error of the mean. T1—basal diet (broilers were fed a diet with neither mycotoxins nor adsorbent added); T2—basal diet + 2.5 g/kg adsorbent; T3—basal diet + 5.0 g/kg adsorbent; T4—AF-contaminated diet + 2.5 g/kg adsorbent; T5—AF-contaminated diet + 5.0 g/kg adsorbent; T6—AF-contaminated diet.

**Table 2:** The effect of MGO-CTS adsorbent and AF-contaminated diets on body feed intake, weight gain, feed conversion ratio, and mortality of broiler chickens.

Parameter	Treatment							
	T1	T2	T3	T4	T5	T6	SEM	*p*-Value
Feed intake (g)								
1 to 21 days	1275.44	1268.12	1278.11	1276.57	1279.01	1274.12	2.206	0.79
22 to 35 days	2064.29 ^a,b^	2046.19 ^a,b^	2182.67 ^a,b^	2238.10 ^a^	2048.57 ^a,b^	1984.52 ^b^	29.782	<0.05
1 to 35 days	3339.73 ^a,b^	3314.31 ^a,b^	3459.78 ^a,b^	3514.70 ^a^	3327.58 ^a,b^	3258.65 ^b^	30.513	<0.05
Weight gain (g)								
1 to 21 days	840.86 ^a^	805.27 ^a,b^	822.71 ^a,b^	827.08 ^a,b^	830.94 ^a^	791.51 ^b^	5.329	<0.05
22 to 35 days	1179.28 ^a^	1183.08 ^a^	1217.67 ^a^	1175.34 ^a^	1176.14 ^a^	1059.36 ^b^	15.269	<0.05
1 to 35 days	2020.14 ^a^	1988.35 ^a^	2022.38 ^a^	1972.41 ^a^	2007.08 ^a^	1850.87 ^b^	17.259	<0.05
Feed conversion ratio (FCR) (g/g)								
1 to 21 days	1.52 ^b^	1.58 ^a,b^	1.55 ^a,b^	1.54 ^a,b^	1.55 ^a,b^	1.61 ^a^	0.0103	<0.05
22 to 35 days	1.75 ^a,b^	1.73 ^b^	1.79 ^a,b^	1.91 ^a^	1.79 ^a,b^	1.88 ^a,b^	0.0229	<0.05
1 to 35 days	1.67 ^b^	1.68 ^b^	1.70 ^b^	1.72 ^a,b^	1.68 ^b^	1.76 ^a^	0.0133	<0.05
Mortality (%)								
1 to 35 days	4	8	4	4	6	10	1.1517	0.387

Data represent mean values of five replicate cages, each with 10 chickens. ^a,b^ Means within a row with no common superscript differ significantly (*p* < 0.05). SEM—standard error of the mean. T1—basal diet (broilers were fed a diet with neither mycotoxins nor adsorbent added); T2—basal diet + 2.5 g/kg adsorbent; T3—basal diet + 5.0 g/kg adsorbent; T4—AF-contaminated diet + 2.5 g/kg adsorbent; T5—AF-contaminated diet + 5.0 g/kg adsorbent; T6—AF-contaminated diet.

**Table 3:** The effect of MGO-CTS adsorbent and AF-contaminated diets on relative organ weight (g/100 g body weight).

Treatment								
Organ Weight (g/100 g Body Weight)	T1	T2	T3	T4	T5	T6	SEM	*p*-Value
Gizzard	1.797	1.652	1.662	1.791	1.831	1.815	0.027	0.236
Liver	2.018	2.202	2.011	1.942	2.138	1.955	0.038	0.292
Heart	0.449	0.466	0.473	0.487	0.487	0.485	0.006	0.489
Pancreas	0.211	0.216	0.200	0.193	0.208	0.198	0.204	0.604
Spleen	0.110 ^b^	0.113 ^b^	0.116 ^b^	0.126 ^a,b^	0.113 ^b^	0.134 ^a^	0.009	<0.05
Kidney	0.173 ^b^	0.200 ^a^	0.198 ^a^	0.194 ^a^	0.191 ^a^	0.205 ^a^	0.004	<0.05
Proventriculus	0.479	0.450	0.454	0.523	0.483	0.475	0.009	0.365
Bursa of Fabricius	0.090	0.110	0.120	0.110	0.100	0.095	0.008	0.467

Values are means of five replicates cages, each with two chickens (*n* = 10). ^a,b^ Means within a row with no common superscript differ significantly (*p* < 0.05). SEM—standard error of the mean. T1—basal diet (broilers were fed a diet with neither mycotoxins nor adsorbent added); T2—basal diet + 2.5 g/kg adsorbent; T3—basal diet + 5.0 g/kg adsorbent; T4—AF-contaminated diet + 2.5 g/kg adsorbent; T5—AF-contaminated diet + 5.0 g/kg adsorbent; T6—AF-contaminated diet.

**Table 4:** The effect of MGO-CTS adsorbent and AF-contaminated diets on biochemical and hematological parameters.

Treatment								
Serum Profile	T1	T2	T3	T4	T5	T6	SEM	*p*-Value
SGOT/AST ^1^ (IU L^−1^)	311.0	281.2	319.5	295.0	282.0	271.2	7.639	0.421
SGPT/ALT ^2^ (IU L^−1^)	6.40 ^a^	4.70 ^a,b^	3.50 ^b^	3.80 ^b^	4.10 ^a,b^	3.20 ^b^	0.259	<0.05
Urea nitrogen (mmol L^−1^)	0.220	0.270	0.300	0.300	0.210	0.240	0.019	0.649
γ-GT ^3^ (IU L^−1^)	18.70	20.20	21.30	21.30	17.40	17.70	0.627	0.267
Alkaline phosphatase (IU L^−1^)	2765.9 ^a^	2114.8 ^a,b^	2766.4 ^a^	1952.1 ^b^	2174.7 ^a,b^	1784.9 ^b^	83.34	<0.05
Cholesterol (mmol L^−1^)	2.960	2.930	3.300	3.310	3.190	3.010	0.060	0.241
Total proteins (g L^−1^)	29.02	27.99	29.59	30.12	27.19	27.07	0.567	0.537
Albumins (g L^−1^)	10.55 ^a^	9.84 ^a,b^	10.31 ^a,b^	9.35 ^b^	9.39 ^b^	9.49 ^b^	0.186	<0.05
Globulins (g L^−1^)	19.07	18.15	19.28	19.07	17.80	17.58	0.413	0.769
Albumins/globulins	0.529	0.548	0.540	0.583	0.530	0.547	0.546	0.330
Hematocrit (%)	27.67	28.10	26.25	28.30	32.00	29.62	3.03	0.546

Values are means of five replicates cages, each with two chickens (*n* = 10). ^a,b^ Means within a row with no common superscript differ significantly (*p* < 0.05). SEM—standard error of the mean. ^1^ SGOT-AST: aspartate aminotransferase. ^2^ SGPT-ALT: alanine aminotransferase. ^3^ γ-GT: γ-glutamyltransferase. T1—basal diet (broilers were fed a diet with neither mycotoxins nor adsorbent added); T2—basal diet + 2.5 g/kg adsorbent; T3—basal diet + 5.0 g/kg adsorbent; T4—AF-contaminated diet + 2.5 g/kg adsorbent; T5—AF-contaminated diet + 5.0 g/kg adsorbent; T6—AF-contaminated diet. ALT—alanine; AST—aspartate.

**Table 5:** Composition of the basal diet.

Ingredient (g/kg Unless Stated Otherwise)	Starter (1 to 21 Days)	Grower (22 to 35 Days)
Ground yellow corn	521.2	460.0
Soybean meal	347.0	249.5
Palm kernel cake (PKE)	50.0	200.0
Dicalcium phosphate	17.0	13.0
Salt (NaCl)	3.0	3.5
Vitamin Premix ^1^	0.5	0.5
Mineral Premix ^2^	1.0	1.0
Corn oil	44.0	60.0
Limestone	11.0	8.0
Choline	0.5	0.5
L-Lysine HCl	3.2	2.5
DL-methionine	1.6	1.5
Total	1000.0	1000.0
Calculated chemical analysis		
Metabolisable energy (Mcal/kg)	2.99	2.94
Crude protein (CP)	208.0	184.0
Fat	63.1	52.2
Fiber	39.0	36.5
Methionine	4.8	4.4
Lysine	13.0	10.2
Calcium	9.2	7.2
Phosphorus	4.2	3.3

^1^ Vitamin premix (per kg premix): folic acid 0.33 g; thiamin 0.83 g; pyridoxine 1.33 g; biotin 03 g; riboflavin 2 g; cyanocobalamin 0.03 g; D-calcium pantothenate 3.75 g; niacin 23.3 g; retinol 2000 mg; cholecalciferol 25 mg; α-tocopherol 23,000 mg IU. ^2^ Mineral premix (per kg premix): iron 100 g; manganese 110 g; copper 20 g; zinc 100 g; iodine 2 g; selenite 0.2 g; cobalt 0.6 g; santoquin 0.6 g.

**Table 6:** Content of MGO-CTS adsorbent added and AFs (AFB_1_, AFB_2_, AFG_1_, and AFG_2_) concentration determined in the diets fed to the six treatment groups.

Treatment	Presence of Adsorbent [Added to Feed (g kg^−1^)]	Presence of Mycotoxin (µg/kg) ^1^				
		AFB_1_	AFB_2_	AFG_1_	AFG_2_	Total AF
T1	No	ND	ND	ND	ND	ND
T2	Yes (2.5 g kg^−1^)	ND	ND	ND	ND	ND
T3	Yes (5.0 g kg^−1^)	ND	ND	ND	ND	ND
T4	Yes (2.5 g kg^−1^)	21.0 ± 0.12	5.0 ± 0.81	12.0 ± 0.16	3.0 ± 0.11	41.0 ± 0.15
T5	Yes (5.0 g kg^−1^)	19.0 ± 0.23	4.0 ± 0.50	11.0 ± 0.51	4.0 ± 0.18	38.0 ± 0.31
T6	No	22.0 ± 0.11	4.0 ± 0.62	8.0 ± 0.30	4.0 ± 0.22	38.0 ± 0.16

^1^ Results are mean values from three replications ± standard deviations. The concentration of AFs in feeds samples was determined by reverse-phase high-performance liquid chromatography (RP-HPLC) equipped C18 analytical column system. ND—not detected.
